# Multi‐camera field monitoring reveals costs of learning for parasitoid foraging behaviour

**DOI:** 10.1111/1365-2656.13479

**Published:** 2021-05-21

**Authors:** Jessica A. C. de Bruijn, Ilka Vosteen, Louise E. M. Vet, Hans M. Smid, Jetske G. de Boer

**Affiliations:** ^1^ Laboratory of Entomology Plant Sciences Group Wageningen University Wageningen The Netherlands; ^2^ Department of Terrestrial Ecology Netherlands Institute of Ecology (NIOO‐KNAW) Wageningen The Netherlands

**Keywords:** associative learning, brassicaceous plant species, cabbage moth, foraging efficiency, large cabbage white butterfly, memory reliability, non‐host, parasitic wasp

## Abstract

1. Dynamic conditions in nature have led to the evolution of behavioural traits that allow animals to use information on local circumstances and adjust their behaviour accordingly, for example through learning. Although learning can improve foraging efficiency, the learned information can become unreliable as the environment continues to change. This could lead to potential fitness costs when memories holding such unreliable information persist. Indeed, persistent unreliable memory was found to reduce the foraging efficiency of the parasitoid *Cotesia glomerata* under laboratory conditions.
2. Here, we evaluated the effect of such persistent unreliable memory on the foraging behaviour of *C. glomerata* in the field. This is a critical step in studies of foraging theory, since animal behaviour evolved under the complex conditions present in nature.
3. Existing methods provide little detail on how parasitoids interact with their environment in the field, therefore we developed a novel multi‐camera system that allowed us to trace parasitoid foraging behaviour in detail. With this multi‐camera system, we studied how persistent unreliable memory affected the foraging behaviour of *C. glomerata* when these memories led parasitoids to plants infested with non‐host caterpillars in a semi‐field set‐up.
4. Our results demonstrate that persistent unreliable memory can lead to maladaptive foraging behaviour in *C. glomerata* under field conditions and increased the likelihood of oviposition in the non‐host caterpillar *Mamestra brassica*. Furthermore, these time‐ and egg‐related costs can be context dependent, since they rely on the plant species used.
5. These results provide us with new insight on how animals use previously obtained information in naturally complex and dynamic foraging situations and confirm that costs and benefits of learning depend on the environment animals forage in. Although behavioural studies of small animals in natural habitats remain challenging, novel methods such as our multi‐camera system contribute to understanding the nuances of animal foraging behaviour.

## Graphical abstract

This article provides new insight on how animals use previously obtained information in naturally complex and dynamic foraging situations and confirms that costs and benefits of learning depend on the environment animals forage in. Furthermore, novel methods such as the multi‐camera system used in this study contribute to understanding the nuances of animal foraging behaviour.

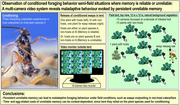

## 1 INTRODUCTION

Use of information to adjust behaviour occurs in the simplest of animals, such as the nematode *Caenorhabditis elegans* (Calhoun et al., 2014), and our current understanding shows that animals employ a fascinating range of behavioural decision‐making mechanisms to optimize foraging behaviour. Natural environments are complex and conditions often change. To forage optimally, animals should anticipate the current state of the environment, but their informational state is generally imperfect (Koops, 2004). Animals therefore need to constantly gather new information to adjust their foraging behaviour to local conditions and thereby improve their foraging efficiency (van Baalen & Hemerik, 2008; Dall et al., 2005).

Learning through prior experience is known to be important in shaping animal foraging behaviour, as it allows an animal to gather, store and use information (Eliassen et al., 2009). Although animals are genetically adapted to respond to cues that have proven to be reliable over many generations, learning allows animals to adapt to local conditions, which is thought to be adaptive in changing environments (van Alphen & Bernstein, 2008; Dall et al., 2005). Foraging behaviour can be altered through different kinds of learning, among which associative learning, where the encounter of a resource (e.g. food) is associated with nearby environmental cues, that is, volatile, tactile or visual information (Honig & James, 1971; Papaj & Lewis, 1993). During this process, local information on resource identity, density, quality and distribution is stored as memory (Eliassen et al., 2009; Hoedjes et al., 2011). Short‐term memory is formed directly after an experience, but fades quickly. More persistent memory forms, such as long‐term memory, can be formed when the resource value (e.g. food quality) or the encounter frequency of the experience is high (Hoedjes et al., 2011; Honig & James, 1971). Both memory forms facilitate temporal adaptation of animal foraging behaviour (Eliassen et al., 2009; Smid & Vet, 2016).

Most studies on associative learning tend to focus on a single learning experience, or repetitive experiences, with a single resource and cue (e.g. Durier & Rivault, 2000; Smid et al., 2007). However, in nature, animals continue to gather and use information from their environment throughout their lifetime. New information can be integrated as additional memories, and previously obtained information needs to be continuously re‐evaluated to validate its reliability in order to maintain a high foraging efficiency, especially in environments with a high degree of within‐lifetime variation. Animals can learn that associated cues do not always reliably predict resource presence and subsequently (temporarily) alter their learned behaviour or continue to use the information when it still has a net benefit (J. A. C. de Bruijn, L. E. M. Vet, H. M. Smid, & J. G. de Boer, manuscript under review; Koops, 2004). The latter may occur when the information is rarely unreliable or the cost of using it is relatively small compared to the benefit when the information turns out to be reliable. Some studies also suggest that animals can be aware of the level of uncertainty in their own knowledge about the current state of their environment (Sulikowski, 2017). For example, noisy miner birds plan their foraging paths in advance when foraging for nectar, but do not when foraging for invertebrate prey that have a less predictable distribution (Sulikowski & Burke, 2010), and honey bees selectively avoid making choices in situations where information is limited (Perry & Barron, 2013).

A subsequent question would be how such fine‐tuning of behaviour, with respect to information reliability, affects an animal's foraging efficiency and ultimately fitness. A high foraging efficiency is expected to improve fitness by increasing lifetime reproductive success, but in most animal species there is no direct link between foraging efficiency and fitness, making it difficult to test this prediction experimentally. However, in insect parasitoids that forage for hosts to lay their eggs, foraging behaviour is directly linked to fitness, and they are therefore ideal model organisms for studies of foraging theory (Thiel & Hoffmeister, 2009; van Baalen & Hemerik, 2008). Parasitoid wasps lay their eggs in or on other organisms, which function as a host for the developing offspring, ultimately resulting in the death of the host (Godfray, 1994). Due to this intimate relationship, hosts have evolved inconspicuousness and mechanisms to kill deposited parasitoid eggs (van Baalen & Hemerik, 2008; Vinson, 1998). This challenges parasitoids to find hosts that are suitable for offspring development. Because of temporal and spatial variation in species composition and host availability, parasitoid foraging behaviour and how it is fine‐tuned by learning are expected to be under strong selection pressure (Thiel & Hoffmeister, 2009).

Parasitoid wasps are well known to employ associative learning to improve their foraging efficiency, where previously associated environmental cues, such as herbivore‐induced plant volatiles, guide females to subsequent plants with hosts (Kruidhof et al., 2015, 2019; Papaj & Vet, 1990). This obtained information can become unreliable when plant species that were previously associated with suitable hosts, now contain unsuitable host stages or non‐host species. Little is known about how such unreliable memories may influence parasitoid foraging behaviour and ultimately fitness. However, a recent laboratory study with the parasitoid *Cotesia glomerata* has shown that persistent unreliable memory, no longer correctly predicting host presence, caused parasitoids to be highly attracted to non‐host‐infested plants and reduced foraging efficiency (de Bruijn et al., 2018).

Here, we extend this study to the field because a controlled laboratory environment is not representative of natural complex and dynamic conditions, under which foraging and learning have evolved (Vet, 2001). Our semi‐field study allowed us to evaluate foraging theory in a natural complex environment, with natural background vegetation and insect communities, at a larger spatial and temporal scale. Assessing foraging efficiency is very challenging in such experiments and has generally been constrained to measuring parasitism levels after a set amount of time (Bukovinszky et al., 2012; De Rijk et al., 2018; Kruidhof et al., 2015). Although this shows the final result of the foraging process, it provides no information on how parasitoids spend their time and energy during foraging, how they interact with their environment (Casas et al., 2003) and how experiences shape their behaviour. We therefore developed a novel multi‐camera set‐up to trace parasitoids during foraging in a complex environment. This approach allowed us to monitor in detail foraging behaviour of *C. glomerata* on host‐ and non‐host‐infested plants. We used this system to test whether persistent unreliable memory, that is, unreliable information acquired during three oviposition experiences spaced in time, leads to decreased foraging efficiency of *C. glomerata*, as observed in the laboratory (Bruijn, Vet, & Smid, 2018). Because we previously found that plant species had a profound effect on conditioned behaviour, we used a reciprocal approach to test our prediction, with two foraging situations where host‐ and non‐host‐infested plant species were switched.

## 2 MATERIALS AND METHODS

### 2.1 Insects

Insect cultures originated from individuals collected in fields of Brussels sprouts near Wageningen. All insects were cultured at the Laboratory of Entomology, Wageningen University, in a climate‐controlled greenhouse at 21 ± 1°C, 50%–70% relative humidity and a L16:D8 photoperiod with both natural and artificial light. Caterpillars of the large cabbage white butterfly, *Pieris brassicae* (Lepidoptera: Pieridae), and the cabbage moth, *Mamestra brassicae* (Lepidoptera: Noctuidae), were reared on Brussels sprouts plants (*Brassicae oleracea* var. gemmifera cultivar Cyrus). *Mamestra brassicae* was only used in experiments as an unsuitable host of the parasitoid *Cotesia glomerata* (Hymenoptera: Braconidae). *C. glomerata* can be considered a generalist as it parasitizes several Pieridae caterpillar species, which are primarily found on brassicaceous plants. Our parasitoid colony was reared on its main host *P. brassicae*. Once *C. glomerata* larvae emerged and cocoons had formed, they were collected and placed in a mesh cage (Bugdorm‐1 Insect rearing cage, 30 × 30 × 30 cm, type DP1000, Megaview Science). Parasitoids were provided with water and honey and kept in a climate cabinet (21 ± 1°C, 50%–70% humidity and L16:D8). For experiments, we used female parasitoids 3 to 5 days after adult emergence.

### 2.2 Plants

We used 4–5‐week‐old brassicaceous plants, *Brassica nigra*, *Sinapis arvensis* and *Brassica oleracea,* for experiments. Plants were watered daily and upright growth was facilitated with a wooden stick (30 cm long, 4 mm diameter). Host‐ and non‐host‐infested plants were prepared as described in detail in Bruijn, Vet, and Smid (2018). Non‐host‐infested plants were infested 48 hr before a field trial by placing five first instar *Mamestra brassicae* caterpillars in the bottom of a clip cage and attaching two of these clip cages to the underside of a leaf. Host‐infested plants were infested 24 hr before a field trial by placing two batches of five first instar *Pieris brassicae* caterpillars on top of a leaf, containing each batch in a clip cage. By the time the experiment started, these caterpillars had chewed through the leaf and were feeding on the underside, where they are also found naturally. *M. brassicae* was allowed to infest the plants twice as long as *P. brassicae* because this results in comparable feeding damage and associated herbivore‐induced plant volatile induction (Bruijn, Vet, & Smid, 2018). Fully expanded straight leaves of similar sizes and at similar heights were selected for infestation. Clip cages were supported with a wooden stick.

### 2.3 Field tent set‐up

**FIGURE 1:**
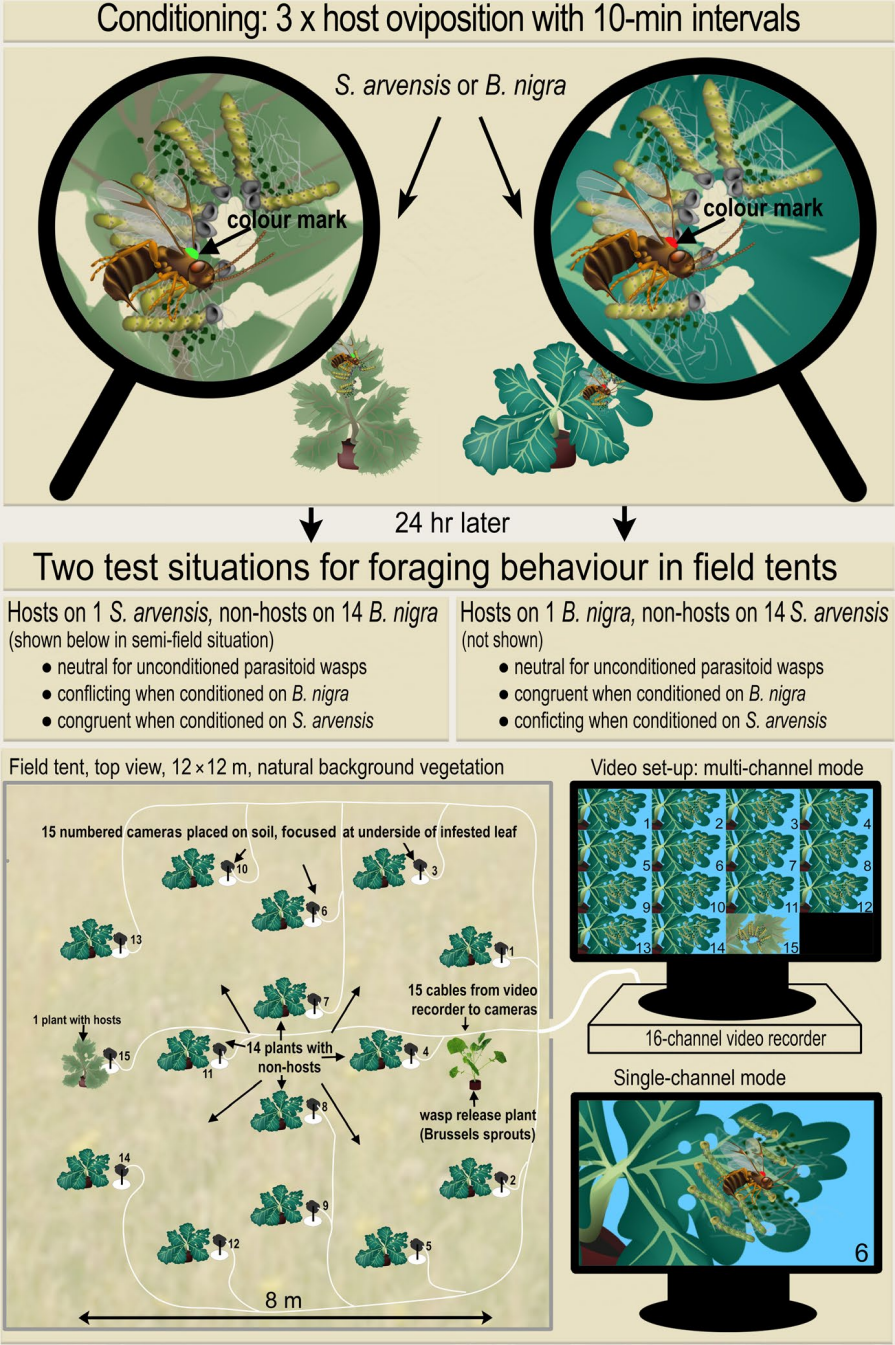
Overview of the experimental approach, including conditioning method of the colour marked *Cotesia glomerata* parasitoids (top panel) and the semi‐field set‐up for the observation of parasitoid foraging behaviour with the multi‐camera set‐up (bottom panels). Colour marked parasitoids were given three spaced oviposition experiences on either a *Brassica nigra* or *Sinapis arvensis* host‐infested plant (top panel) or were kept unconditioned (not shown). They were released in the two test situations 24 hr after conditioning, as described in the middle panels. The bottom panels show the layout of the field tent, with the locations of the host‐ and non‐host‐infested plants, their associated video cameras, their connection to the video recorder and the display of the 15 video channels, with the multi‐channel mode to observe parasitoid activity on all plants and the single‐channel mode for a more detailed view. See text for a more detailed description

To observe foraging behaviour of parasitoids, four large mesh tents (12 m × 12 m × 2.5 m) were placed in a field near the campus of Wageningen University. The field consisted of natural grass and herb vegetation, with various species of *Poaceae*, *Juncaceae* and *Cyperaceae* and plants such as *Bellis perennis*, *Rumex acetosa* and *Trifolium pratense*. We confirmed that brassicaceous plants were absent during our field trial, but such plants can naturally occur in Dutch grasslands and form natural habitats where *C. glomerata* forages. Within each tent, 16 test plants were positioned in an 8 × 8 m matrix, presented in Figure 1. On one side of the tent a single host‐infested plant was placed, on the opposite side, we positioned the parasitoid release plant (always an uninfested *B. oleracea* plant). All other positions in the matrix were occupied by non‐host‐infested plants. This layout ensured that parasitoids would encounter several non‐host‐infested plants after their release, before reaching the host‐infested plant. Pots with infested plants were placed on top of the soil within the dense natural vegetation. Vegetation height was adjusted to the height of the infested plants and kept at approximately 30 cm through trimming with hedge shears every 2 weeks.

Since foraging behaviour of parasitoids may be influenced by the plant species used, two reciprocal foraging situations were created. Foraging situation 1 consisted of 14 non‐host‐infested *S. arvensis* plants, a single host‐infested *B. nigra* plant and an uninfested *B. oleracea* release plant. Foraging situation 2 consisted of 14 non‐host‐infested *B. nigra* plants, a single *S. arvensis* host‐infested plant and a *B. oleracea* release plant (see Figure 1). After placing plants in the field, clip cages were removed, cotton wool was wrapped around the stalk of the leaf to prevent caterpillars from dispersing to other leaves and the number of caterpillars on each plant was checked. Missing caterpillars were replaced by caterpillars of the same age to ensure that 10 caterpillars were present on each plant at the start of the experiment. Below each infested plant, a camera was placed that was focussed at the underside of the damaged leaf with caterpillars. Wooden sticks of 15 cm and 30 cm were used when necessary to support the horizontal position of the leaf to allow for a good view of the lower leaf surface. Each week, both foraging situations were tested once on different days, with the order of the two foraging situations randomized per week. Plants and cameras were removed from the tent after each trial, and each tent was used only once every 2 weeks. Although we expected that parasitoids would die within a week because no food was provided and very few flowering plants were present, yellow sticky traps (Horiver^®^, Koppert Biological Systems, Berkel en Rodenrijs, the Netherlands) were placed in the tent after each recording to capture remaining parasitoids.

### 2.4 Colour marking

To ensure that individual *C. glomerata* females could be recognized during the field trials, parasitoids were given one of 12 colour markings. We used glossy enamel paints (Revell GMBH, Germany) in the colours white (#4), yellow (#12), red (#31), orange (#30), blue (#50) and green (#61), which were applied in different one to three dot patterns to create 12 different colour markings, randomly assigned to the different treatments of the parasitoids. Due to the fast‐drying properties of the paint, a thinner (Revell Thinner, Revell GmbH & Co. KG) was used to maintain a proper consistency of the paint. For colour marking, females were captured individually in a glass vial and briefly placed on ice to anaesthetize them, after which an immobilized individual was placed under a microscope and the colour pattern was applied on top of the thorax (see Figure 1, top panel) with a fine nylon brush. After colour application, the parasitoid was transferred to a small mesh cage (Bugdorm type 41,515, 17 × 17 × 17 cm, Megaview Science) with water and honey and kept in a climate cabinet until conditioning the following day. We did not observe signs of increased mortality of *C. glomerata* due to application of these colour marks.

### 2.5 Conditioning procedure

Parasitoids were either kept unconditioned or were conditioned with three oviposition experiences spaced by 10 min to form persistent memory, as described in Bruijn, Vet, and Smid (2018) and depicted in Figure 1. Plants for conditioning were infested 24 hr prior with 200–300 first instar *P. brassicae* caterpillars, distributed in groups of 50 over the leaves of either a *B. nigra* or a *S. arvensis* plant. Colour marked females were allowed to oviposit in host caterpillars on a leaf of either an infested *B. nigra* or *S. arvensis* plant, allowing for the formation of an association between oviposition and the plant's volatiles. A female parasitoid was first captured in a glass vial and then transferred to the leaf with hosts. The parasitoid was allowed to oviposit once in a caterpillar and then recaptured in a vial. This procedure was repeated twice at 10‐min intervals. Conditioned parasitoids were kept with honey and water in a small cage that was placed in a climate cabinet until the start of the field trial the following day.

For parasitoids conditioned on *B. nigra*, foraging situation 1 (one *B. nigra* host plant and 14 *S. arvensis* non‐host plants) was congruent with the information they had obtained, that is, they had reliable memory, while for parasitoids conditioned on *S. arvensis* it conflicted with what they had learned, that is, they had unreliable memory. Foraging situation 2 (one *S. arvensis* host plant and 14 *B. nigra* non‐host plants) conflicted with the information obtained by parasitoids conditioned on *B. nigra*, while for parasitoids conditioned on *S. arvensis* it was congruent. For unconditioned parasitoids both foraging situations were neutral. This reciprocal approach resulted in six different treatments: the three different experience types (conflicting, congruent and unconditioned), which were tested in each of the two foraging situations.

### 2.6 Video set‐up

In order to observe the activity of the parasitoids assigned to these six treatments, we used a multi‐camera set‐up (Cabled 16‐dome‐system PLUS, Bascom cameras bv, Nieuwegein, The Netherlands) that consisted of 16 dome camera's and a 16‐channel hard disc recorder (recorder type PR16K, with a 1 TB hard drive) for data and power transfer (Power over Ethernet, PoE). The cameras (type bsm‐pd20) had manual focusing and zoom function (92° to 28°) and a resolution of 2,048 × 1,536 pixels. A UTP cable connected each camera to the hard disc recorder for data transfer and power supply (Figure 1). The video recorder and a 24‐inch screen (Phillips 221B) were placed on a table outside the tent. The recorder was connected to the screen via a 16 m long VGA cable, to allow bringing the monitor inside the tent for manual focusing of each camera during set‐up. During each trial, we used 15 cameras that were simultaneously displayed as 15 live view video channels on the screen. An enlarged, single camera view window could be used when parasitoid activity was detected by the observer. Since the cameras were directed towards the sky, there was substantial contrast in the recordings, which reduced the saturation of the colour codes on the parasitoids, making it difficult to distinguish these codes reliably. Parasitoid identification was therefore confirmed by an observer inside the tent, upon request of the observer at the screen.

### 2.7 Behavioural observations

Each experimental day, 12 *C. glomerata* females were transferred from a cage to a glass vial (28.5 × 95 mm): four conflictingly and four congruently conditioned parasitoids and four unconditioned parasitoids. This vial was placed directly next to the stem of the release plant. Parasitoids were left to acclimatize for 5 min, after which they were released and video recording was started. Within 30 min all parasitoids had left the vial. Parasitoids could forage for a maximum of 5 hr, during which parasitoid presence on host and non‐host plants was monitored continuously on the screen. As soon as a parasitoid was observed to land on a plant (as observed in one of the 15 video channels on the monitor outside the tent), its colour marking was checked by the observer in the tent and the time of this first sighting and the associated video channel were noted. Parasitoids had completed the foraging experiment when they found the host caterpillars. Thereafter they were captured and removed from the tent to prevent them from disturbing and parasitizing all caterpillars and to avoid any disturbance of the landings of subsequently arriving parasitoids. After 5 hr, the recording was terminated. All trials were done between 7:00 and 17:30, where we planned the 5 hr recording during favourable weather conditions, that is, 18–28°C and no rain. The experiment ran between May and September 2018, with each foraging situation tested eight times, resulting in 32 parasitoids per treatment.

### 2.8 Video data collection and processing

Video files were retrieved from the recorder and stored on a hard disc, after which behavioural data were manually retrieved from these recordings with Windows Media Player (version 12.0.7601.24312, © 2009 Microsoft Corporation). Records on the time of first sighting and the associated video channels were used as a starting point to collect data from the video recording on the foraging behaviour of each individual parasitoid. With backtracking, we first determined when the parasitoid landed on the plant for the first time. Since parasitoids would frequently depart from the plant and then land again within a few seconds, often hovering around the infested leaf, backtracking was done until the parasitoid was not seen for 2 min. After the first landing, forward tracking was applied to determine all subsequent arrival and departure times to the individual non‐host plants and we verified whether, and how often, parasitoids oviposited in the non‐host. If the parasitoid was not seen for 2 min after the last sighting, it was considered to have left the plant. An arrival and subsequent departure were considered a non‐host plant visit when the arrival and subsequent departure times differed by more than 1 s, otherwise it was considered a jump. These jumps were not used in data analyses. Occasionally, more than one parasitoid was observed foraging on a single infested leaf, which led to a confusion of parasitoid identity in a few cases. If there was any doubt about the identity of the parasitoid, its colour code was reported as unknown, and this observation was not used for further analyses.

These collected data were used, in combination with presence/absence data on whether released parasitoids started to forage and whether they found the host, to determine values of 14 different foraging parameters per individual parasitoid, described in Table 1. For the foraging parameter ‘time to host’ (FP 3), parasitoids were assigned the maximum time of 5 hr when they did not manage to find the host during the recording. In reality, these parasitoids may have needed more than 5 hr to find the host, and we therefore analysed this parameter with and without these unsuccessful parasitoids because including them better reflects the difficulty of finding the host.

**TABLE 1:** Description of the 14 foraging parameters (FP) and the type of data they consisted of (continuous, count or binary)

Code	Foraging parameter	Type	Description
FP 1	Response	Binary	Whether a released parasitoid was observed foraging
FP 2	Host found	Binary	Whether a foraging parasitoid found the host
FP 3	Time to host	Continuous	The time from the start of the recording, that is, time of release, until the parasitoid landed on the host plant
FP 4	Time until first landing	Continuous	The time from the start of the recording until the first landing on an infested plant
FP 5	Foraging time	Continuous	The time the parasitoid was foraging, calculated by subtracting time until first landing (FP 4) from time to host (FP 3)
FP 6^a^	NH plant visit residence time	Continuous	The residence time of a single non‐host plant visit, calculated by subtracting a departure time from the prior arrival time
FP 7^a^	NH plant residence time	Continuous	The time a parasitoid spent on an individual non‐host plant, where all revisits were summed
FP 8	Total NH plant residence time	Continuous	The sum of all non‐host plant visit residence times of a parasitoid
FP 9^a^	Inter‐patch time	Continuous	The time parasitoids used to move from one non‐host plant, that is, patch, to another, where revisits to the same plant were ignored
FP 10^a^	Intra‐patch time	Continuous	The time it took to revisit the same non‐host plant
FP 11	NH plant visits	Count	The cumulative number of visits to all non‐host plants
FP 12	NH plants visited	Count	The number of individual non‐host plants that were visited (out of 14)
FP 13	NH oviposition	Binary	Whether a foraging parasitoid oviposited in a non‐host
FP 14	NH ovipositions	Count	The number of times a parasitoid oviposited in the non‐host, 0 included

^a^ Foraging parameters FP 6, FP 7, FP 9 and FP 10 were based on an average time per individual parasitoid.

### 2.9 Statistics

R version 3.5.0 (R Core Team, 2018) was used for analyses. We used several types of models, which included parasitoid experience as a fixed factor, and a nested random factor to account for day variation in the different tents (day nested within tent) when possible. Separate models were made for the two foraging situations because parasitoid behaviour was clearly context dependent (see Results). Binary data (FP 1, 2, 13) were analysed using generalized linear mixed‐effect models (glmer models, lme4 package, Bates et al., 2014). Data on unconditioned parasitoids (neutral) were only used in the binary response models of FP 1 and FP 2 because the number of parasitoids that responded was too low to analyse the other parameters (see below).

Time to host (FP 3) and time until first landing (FP 4) were analysed using survival analysis with Cox's proportional hazard models with frailty (survival package, Therneau and Lumley (2015). Data were censored when parasitoids did not reach the host (FP 3) or when they were not seen landing on an infested plant (FP 4) within 5 hr. Linear mixed‐effect models were used to analyse continuous data on foraging time (FP 5, both with and without unsuccessful parasitoids), NH plant visit residence time (FP 6), NH plant residence time (FP 7), total NH plant residence time (FP 8) and inter‐ (FP 9) and intra‐patch (FP 10) times (nlme package, Pinheiro et al., 2014). Data were log‐transformed when residuals were not normally distributed. Count data on NH plant visits (FP 11) were analysed using a glmer model with a Poisson distribution and the number of NH plants visited (FP 12) was analysed with a glmer model with a negative binomial distribution to correct for overdispersion. Data on NH ovipositions (FP 14) were zero‐inflated and therefore analysed with a zero‐inflated model with a negative binomial distribution.

## 3 RESULTS

As expected, the two reciprocal foraging situations caused parasitoids to forage in different ways. In foraging situation 1, with the 14 *S. arvensis* non‐host‐infested plants and the one *B. nigra* host‐infested plant, only 23% of the released parasitoids were observed to start foraging on the infested plants (FP 1). In foraging situation 2, with the 14 *B. nigra* non‐host‐infested plants and the one *S. arvensis* host‐infested plant, we observed 42% of parasitoids foraging.

Parasitoid response (FP 1) was not significantly influenced by parasitoid experience, although the proportion of unconditioned foraging females (neutral) was lowest in both foraging situations. In foraging situation 1, 22% of parasitoids given conflicting information started to forage, 31% with congruent information and only 16% of neutral parasitoids (*X* ^2^ = 2.240, *p* = 0.326). In foraging situation 2, 53% of parasitoids given conflicting information started to forage, 41% with congruent information and only 32% of neutral parasitoids (*X* ^2^ = 3.203, *p* = 0.202). Of those parasitoids that started foraging, approximately 50%–60% of parasitoids found the host (FP 2), except in foraging situation 1, where 80% (4 out of 5) of neutral parasitoids found the host. In both foraging situations, the proportion of parasitoids that found the host was not influenced by parasitoid experience (FP 2, foraging situation 1: *X* ^2^ = 0.109, *p* = 0.947; foraging situation 2: *X* ^2^ = 1.151, *p* = 0.563).

**FIGURE 2:**
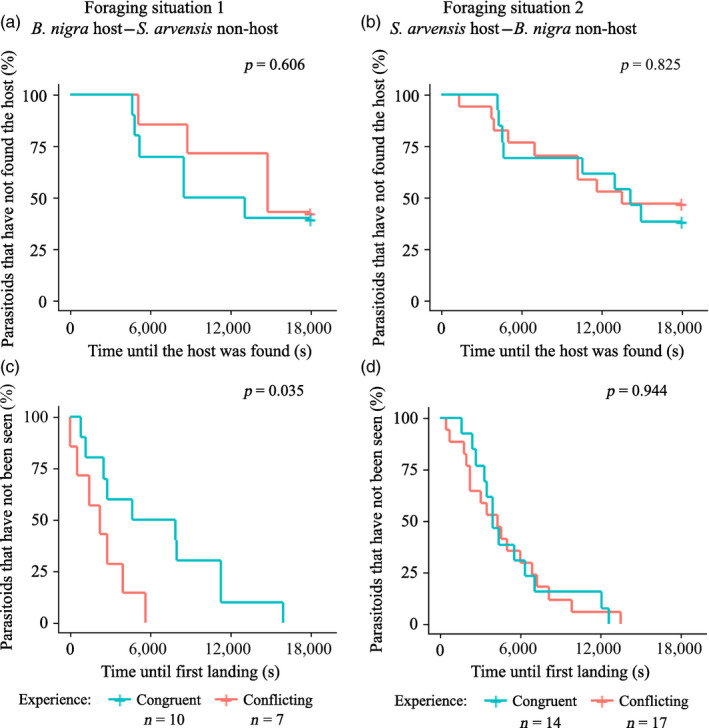
Survival plots of the fraction of *Cotesia glomerata* parasitoids with congruent and conflicting memory that have not found the host within 5 hr (FP 3) in foraging situation 1 (a) and foraging situation 2 (b), and the fraction of parasitoids that have not been seen, that is, have not landed on an infested plant (FP 4) in foraging situation 1 (c) and foraging situation 2 (d). These parasitoids foraged in a semi‐field set‐up with *Pieris brassicae* (host) and *Mamestra brassicae* (non‐host) infested plants. *p*‐values are based on Cox's proportional hazard models with frailty and indicate whether the survival curves of parasitoids with conflicting and congruent memory are significantly different

**FIGURE 3:**
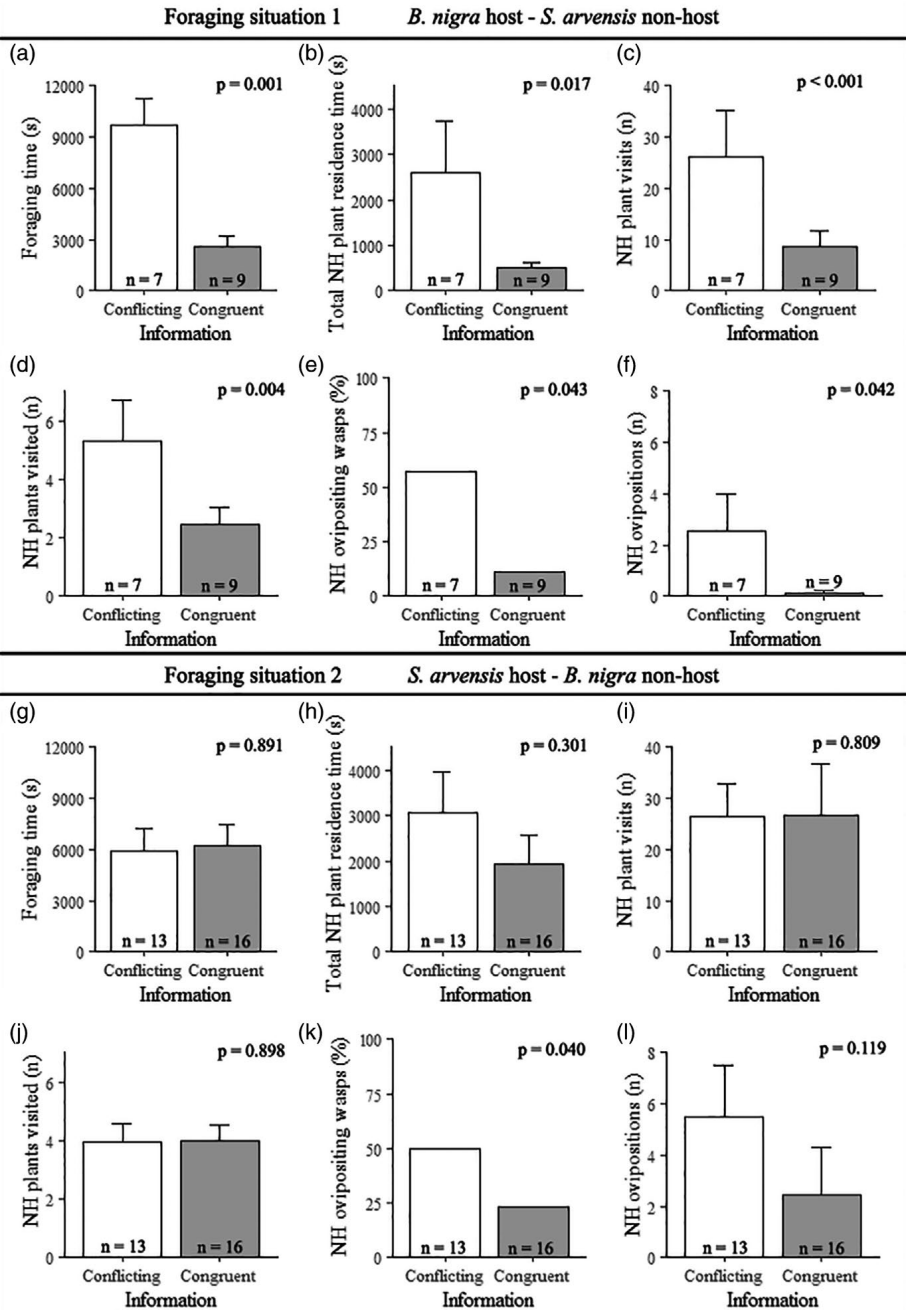
The foraging behaviour of *Cotesia glomerata* parasitoids with conflicting and congruent memory on and around host‐ and non‐host‐infested plants. Various behavioural parameters were measured in foraging situation 1 (a–f) and foraging situation 2 (g–l). Panels show how long parasitoids foraged (FP 5, a/g), how much time they spent in total on the non‐host (NH) plants (FP 8, b/h), how many visits parasitoids made to NH plants (FP 11, d/j), how many NH plants were visited (FP 12, c/i), the percentage of parasitoids that oviposited in the non‐host (FP 13, e/k) and the number of ovipositions in the non‐host (FP 14, f/l). Average values are shown with error bars representing the *SE*, except for panels e and k, which show overall percentages. Numbers of wasps per treatment are indicated inside bars. *p*‐values show whether the behavioural parameter was significantly different between parasitoids with conflicting and congruent memory

In foraging situation 1, we observed clear differences in foraging behaviour of parasitoids given conflicting and congruent information (Figures 2 and 3), although parasitoids given conflicting or congruent information did not differ in how long it took them to find the host on *B. nigra* (FP 3, Figure 2a). Parasitoids given congruent information started foraging significantly later (FP 4, Figure 2c) and their time spent foraging was less than a third of that of parasitoids given conflicting information (FP 5, Figure 3a). Foraging time was also significantly lower for parasitoids given congruent information, when individuals that did not find the host were excluded (FP 5, *F* = 178.278, *p* = 0.006). Interestingly, parasitoids given conflicting information spent more than five times longer on the non‐host‐infested *S. arvensis* plants than parasitoids with congruent information (FP 8, Figure 3b). They visited twice the number of non‐host plants (FP 11, Figure 3c) and made three times more visits to non‐host plants (FP 12, Figure 3d). However, NH plant visit residence time (FP 6, *F* = 0.135, *p* = 0.724) and NH plant residence time (FP 7, *F* = 2.332, *p* = 0.171) did not differ between parasitoids with conflicting and congruent information. On non‐host‐infested plants, parasitoids with conflicting information were five time more likely to oviposit in the non‐host (FP 13, Figure 3e) and they oviposited significantly more often (FP 14, Figure 3f) than parasitoids given congruent information. None of the four unconditioned parasitoids that started foraging oviposited in the non‐hosts. The conflicting and congruent groups did not differ with respect to inter‐patch time (FP 9, *F* = 0.079, *p* = 0.790) and intra‐patch time (FP 10, *F* = 0.151, *p* = 0.711).

In foraging situation 2 (Figures 2 and 3), parasitoids given conflicting and congruent information took equally long to find the host on the infested *S. arvensis* plant (FP 3, Figure 2b). Both groups started foraging around the same time (FP 4, Figure 2d) and did not differ in their foraging time (FP 5, Figure 3g). Parasitoid experience did not influence the number of NH plant visits (FP 11, Figure 3j) and number of NH plants visited (FP 12, Figure 3i), nor did it influence NH plant visit residence time (FP 6, *F* = 2.204, *p* = 0.153), NH plant residence time (FP 7, *F* = 1.268, *p* = 0.273) and total NH plant residence time (FP 8, Figure 3h). Furthermore, the two groups also did not differ in inter‐patch time (FP 9, *F* = 0.420, *p* = 0.526) and intra‐patch time (FP 10, *F* = 0.529, *p* = 0.476). Parasitoids given conflicting information were, however, twice as likely to oviposit in the non‐host than parasitoids given congruent information (FP 13, Figure 3k), but the number of ovipositions did not differ (FP 14, Figure 3l) and was high in both groups. Although not included in the binary non‐host oviposition model (FP 13), 50% of the unconditioned parasitoids (5 of 10 individuals) oviposited in a non‐host.

## 4 DISCUSSION

Our multi‐camera system provided detailed insight on the foraging behaviour of *Cotesia glomerata* in relation to information reliability in a complex natural environment. For the first time, we show that persistent unreliable memory influences foraging behaviour of a parasitoid under realistic field conditions, confirming our laboratory findings (Bruijn, Vet, & Smid, 2018). Furthermore, we found that the effect of persistent unreliable memory can depend on the foraging situation, that is, the plant species containing host and non‐host caterpillars, and that persistent unreliable memory can stimulate non‐host oviposition on the conditioned plant species. In foraging situation 1, where a single *Brassica nigra* plant was infested with *Pieris brassicae* host caterpillars and 14 *Sinapis arvensis* plants were infested with *Mamestra brassicae* non‐host caterpillars, parasitoids with persistent unreliable memory spent more time foraging on the non‐host plants. These parasitoids encountered a foraging situation that conflicted with their memory, which led to a higher number of non‐host plants visited and more non‐host plant visits compared to parasitoids with persistent reliable memory that encountered a congruent foraging situation. Furthermore, five times as many parasitoids with persistent unreliable memory oviposited in the non‐host, and each parasitoid also oviposited more often in these non‐hosts, than parasitoids with persistent reliable memory. Persistent memory, formed by three spaced host oviposition experiences on *S. arvensis* plants, thus proved costly to *C. glomerata* in terms of time and eggs spent when foraging in an environment where only non‐host caterpillars were present on these *S. arvensis* plants. In the reciprocal foraging situation (2), we did not observe time‐related effects of persistent unreliable memory, but we did observe that parasitoids with persistent unreliable memory were more prone to oviposit in the non‐hosts, as in foraging situation 1. The lack of time‐related costs in foraging situation 2 suggests that some aspects of persistent unreliable memory depend on the plant species used for conditioning and testing.

Despite the clear effects of memory reliability on foraging behaviour in our trials, it remains difficult to interpret how foraging efficiency is influenced. When it is defined as the time needed to find a suitable host (from parasitoid release to host found), our results suggest that information reliability does not affect foraging efficiency because parasitoids with conflicting or congruent information did not differ in this respect. However, our multi‐camera system revealed clearly that memory reliability had a significant impact on how parasitoids spent their time during foraging. Parasitoids given persistent reliable memory started foraging later on the non‐host plants compared to parasitoids given persistent unreliable memory. Even with our advanced multi‐camera set‐up, we have no information on how these parasitoids spent their time until they were first observed and we do not know whether they were investing time in finding hosts until then. In laboratory studies, initially unresponsive parasitoids are generally excluded when they do not start foraging within 5 min (Bruijn, Vet, & Smid, 2018; Geervliet et al., 1998). In our field trials, discrimination between actively foraging and initially inactive female parasitoids was not possible, illustrating the challenge of monitoring parasitoid behaviour under natural conditions. Nevertheless, parasitoids with persistent reliable memory were less active on and around non‐host‐infested plants and were less likely to oviposit in *M. brassicae* than parasitoids with persistent unreliable memory. Furthermore, if we consider only the time between the first landing on an infested plant and the arrival on the host‐infested plant, effects of information reliability on foraging efficiency are clear because parasitoids with persistent unreliable memory spent more time foraging until the host was found.

Plant species, in particular the plant species infested with non‐host caterpillars, played an important role when considering the effect of information reliability on parasitoid foraging behaviour, underlining the context dependency of our findings. In foraging situation 1, it appears that parasitoids with persistent reliable memory (conditioned on *B. nigra* plants) initially delayed foraging on the non‐host‐infested *S. arvensis* plants because the volatiles of these plants did not match with what they had learned. On the other hand, parasitoids given persistent unreliable memory (conditioned on *S. arvensis*) were directly attracted to the non‐host‐infested *S. arvensis* plants because they had associated host presence with *S. arvensis* volatiles. In foraging situation 2, however, parasitoids of both groups were attracted to the non‐host‐infested *B. nigra* plants, even when they were conditioned on *S. arvensis*. This indicates that the volatiles of these plants were highly attractive to *C. glomerata*, irrespective of the plant species on which they had previously gained oviposition experience. We suggest that in this situation the effect of the previous oviposition experience may be overruled by the high attractiveness, or detectability, of volatiles emitted by *B. nigra* plants infested with non‐host caterpillars. This plant species‐specific behaviour corroborates previous findings with *C. glomerata* (Bruijn, Vet, & Smid, 2018), which is known to show differences in innate attraction to the plant volatiles of brassicaceous plant species and cultivars (Geervliet et al., 1996; Poelman et al., 2009). It is also possible that the preparedness of parasitoids to learn the volatiles of the two plant species differs (Dunlap & Stephens, 2016; Raine & Chittka, 2005; Smid & Vet, 2016). The association formed with *B. nigra* might have led to a stronger behavioural response to this plant species compared to the association formed with *S. arvensis*. Our results suggest that the costs of conflicting information depend on the strength of the innate attraction to the plant species that carries the non‐hosts. If innate attraction is weak, parasitoids with conflicting information show a stronger attraction to non‐host‐infested plants than parasitoids with congruent information (foraging situation 1). If innate attraction to the non‐host‐infested plant species is strong, all parasitoids are attracted to these plants, irrespective of their previous experience (foraging situation 2). Further experiments with different plant species should reveal whether conflicting information is costly in terms of foraging time spent on non‐host‐infested plants in most situations, or whether our findings are an exception.

Our experiment, and particularly the use of multiple video cameras, revealed that persistent unreliable memory increased the tendency of *C. glomerata* to oviposit in the non‐host *M. brassicae*. In both foraging situations parasitoids with persistent unreliable memory were two to five times more likely to oviposit in the non‐host caterpillars compared to parasitoids with reliable memory and parasitoids did so significantly more often in foraging situation 1. It is difficult to compare this to the behaviour of unconditioned parasitoids because very few unconditioned parasitoids started foraging in our study. This can be explained by a generally much lower motivation to start foraging (parasitoid response) in unconditioned compared to conditioned parasitoids (Bleeker et al., 2006). Non‐host oviposition was not observed among the four unconditioned parasitoid individuals that started foraging in foraging situation 1, while 5 of 10 foraging individuals oviposited in a non‐host in foraging situation 2. Although acceptance of this non‐host by *C. glomerata* has been described in several laboratory studies (Bruijn, Vet, & Smid, 2018; Bukovinszky et al., 2012; Vosteen et al., 2019), this is the first study that clearly shows that associative learning influences the frequency of this seemingly maladaptive behaviour. We were not able to assess the impact of multiple non‐host ovipositions on subsequent foraging behaviour and fitness of *C. glomerata*, since our trial ended after 5 hr. Subsequent foraging behaviour may be altered by frequent non‐host ovipositions, possibly leading to avoidance of these plants. Our finding that most parasitoids were successful in finding the host‐infested plant before the experiment ended, could even be a result of this. Alternatively, this could be an artefact of containing the parasitoids in a tent. Based on our findings, we predict that persistent unreliable memory may reduce the fitness of *C. glomerata* due to the high number of non‐host ovipositions, leading to lower egg loads and energy spent during foraging on plants infested with non‐hosts as well as associated risks of predation. Ultimately, the (fitness) costs of unreliable memories will depend on species and context; in the case of *C. glomerata* on plant and caterpillar species composition and distribution in the wider environment. Longer term experiments at a larger scale and more sophisticated tracking of animal behaviour are required to gain more insight in these costs and the conditions under which they occur.

Indeed, the application of our multi‐camera set‐up can be seen as a first step in the development of more advanced methods to track the behaviour of small animals, such as insects, in the field. The use of cameras, computers and imaging analysis for quantitative studies on insect behaviour has become more widespread in recent years and new techniques are being developed rapidly (Bruijn, Vet, & Smid, 2018; Cholé et al., 2015; de Bruijn, et al. 2018; Gernat et al., 2018; Jiang et al., 2016; Manoukis & Collier, 2019; Reza et al., 2013; Spitzen et al., 2013; Zhou et al., 2015). The system we used could be further improved by including technologies such as QR code tags and smart cameras that can automatically detect these tags (Gernat et al., 2018), and advanced tracking software that can recognize and track small animals despite environmental disturbances, such as wind. Ideally, future technological developments will lead to methods that allow for continuous monitoring of all sorts of animals and their behaviour in the field, with detailed recordings of their interactions with various organisms in their environment and automated extraction of data from video files. For example, in the present experiment, this could have revealed how parasitoids spent their time until they were first observed on a non‐host‐infested plant.

Overall, we conclude that our approach provided detailed insight in the foraging behaviour of *C. glomerata* in relation to information reliability in a natural foraging situation. We demonstrated that persistent memory, containing unreliable information, can affect the foraging behaviour of *C. glomerata*, and result in (fitness) costs. These effects were context specific, since volatiles of highly attractive plant species could overrule the effect of information reliability. Indeed, parasitoid attraction to the non‐host‐infested plants and subsequent non‐host acceptance behaviour after associative learning are specific to the conditioned plant species and not a general effect of learning. To increase our understanding on how learning shapes animal foraging behaviour in nature, future research should be focussed on the effects of information reliability and its context‐dependent effects. The use of more advanced tracking methods to achieve this goal is highly recommended.

## AUTHORS' CONTRIBUTIONS

The idea for a semi‐field experiment was suggested by J.G.d.B., H.M.S. and L.E.M.V.; J.A.C.d.B. designed the study with H.M.S.; Suggestions for the design were provided by L.E.M.V. and I.V.; J.A.C.d.B. organized the experiment and conducted it with H.M.S. and I.V.; J.A.C.d.B. extracted the data from the video files, defined behavioural parameters, analysed the data and made graphs.; J.A.C.d.B. interpreted the data alongside H.M.S., L.E.M.V. and I.V.; H.M.S. made the illustration of the set‐up and the graphical abstract.; J.A.C.d.B. wrote and revised the manuscript with J.G.d.B., H.M.S., L.E.M.V. and I.V. All authors approved the submitted version of the manuscript.

## Data Availability

Data associated with the manuscript are archived in Dryad Digital Repository https://doi.org/10.5061/dryad.905qfttk6 (de Bruijn, 2021).
